# Mobility of charge carriers in self-assembled monolayers

**DOI:** 10.3762/bjnano.10.235

**Published:** 2019-12-11

**Authors:** Zhihua Fu, Tatjana Ladnorg, Hartmut Gliemann, Alexander Welle, Asif Bashir, Michael Rohwerder, Qiang Zhang, Björn Schüpbach, Andreas Terfort, Christof Wöll

**Affiliations:** 1 Institute of Functional Interfaces (IFG), Karlsruhe Institute of Technology (KIT), Campus Nord, 76344 Eggenstein-Leopoldshafen, Germany; 2 Max-Planck-Institut für Eisenforschung GmbH, 40237 Düsseldorf, Germany; 3 Thyssenkrupp Bilstein GmbH, Herner Str. 299, 44809 Bochum, Germany; 4 Department of Chemistry, Institute of Inorganic and Analytical Chemistry, Goethe-University, 60438 Frankfurt, Germany

**Keywords:** conducting atomic force microscopy, lateral charge transport, nanografting, organic semiconductor, self-assembled monolayer

## Abstract

We present a new approach to study charge transport within 2D layers of organic semi-conductors (OSCs) using atomic force microscopy (AFM)-based lithography applied to self-assembled monolayers (SAMs), fabricated from appropriate organothiols. The extent of lateral charge transport was investigated by insulating pre-defined patches within OSC-based SAMs with regions of insulating SAM made from large band gap alkanethiolates. The new method is demonstrated using a phenyl-linked anthracenethiolate (PAT), 4-(anthracene-2-ylethynyl)benzyl thiolate. *I*–*V* characteristics of differently shaped PAT-islands were measured using the AFM tip as a top electrode. We were able to determine a relationship between island size and electrical conductivity, and from this dependence, we could obtain information on the lateral charge transport and charge carrier mobility within the thin OSC layers. Our study demonstrates that AFM nanografting of appropriately functionalized OSC molecules provides a suitable method to determine intrinsic mobilities of charge carriers in OSC thin films. In particular, this method is rather insensitive with regard to influence of grain boundaries and other defects, which hamper the application of conventional methods for the determination of mobilities in macroscopic samples.

## Introduction

Charge transport in organic semiconductors plays a central role in the field of molecular electronics [1]. In addition to hopping transport of charge carriers, electrons or holes, within molecular solids [2], for some materials also a band-like transport has been proposed [3]. Previous work showed that the charge carrier mobilities are highly dependent on the structural quality of the material [4]. Domain boundaries, contaminations and defects have a pronounced, negative effect on charge carrier mobility. This fact calls for measurements on low-defect density samples, preferentially macroscopic single crystals, to determine the intrinsic mobilities. This approach, however, is difficult due to the challenge of production and procurement of highly ordered, very pure crystals of macroscopic dimensions with very low defect density, and accordingly only few measurements of such type were reported [5–8].

Here, we obtain information on intrinsic mobilities, the main figure of merit of OSC materials, using an approach where measurements are carried out for structurally well-defined self-assembled monolayers (SAMs) [9], fabricated from appropriate organothiols. A very large number of organothiolates have been found to form monolayers of high structural quality on Au substrates [10], with a pronounced stability resulting from the formation of a strong S–Au bond grafting the thiolates to the substrate. In selected cases, very high structural quality has been reported, with ordered domains in the size range of several 100 nm [11]. Previous research into the electron transfer within SAMs focused on the vertical charge transport through individual molecules of aromatic SAMs by using conductive atomic force microscope (c-AFM) [12–15] and scanning tunneling microscope (STM) techniques [16–17]. Using these methods, current–voltage (*I*–*V*-) curves on the SAM-forming organothiolates has been determined in a number of cases. All these works focused on the vertical transport through SAMs and only few papers have been published where the lateral transport within SAMs caused by intermolecular charge transfer parallel to the surface has been discussed [18–24].

**Figure 1:**
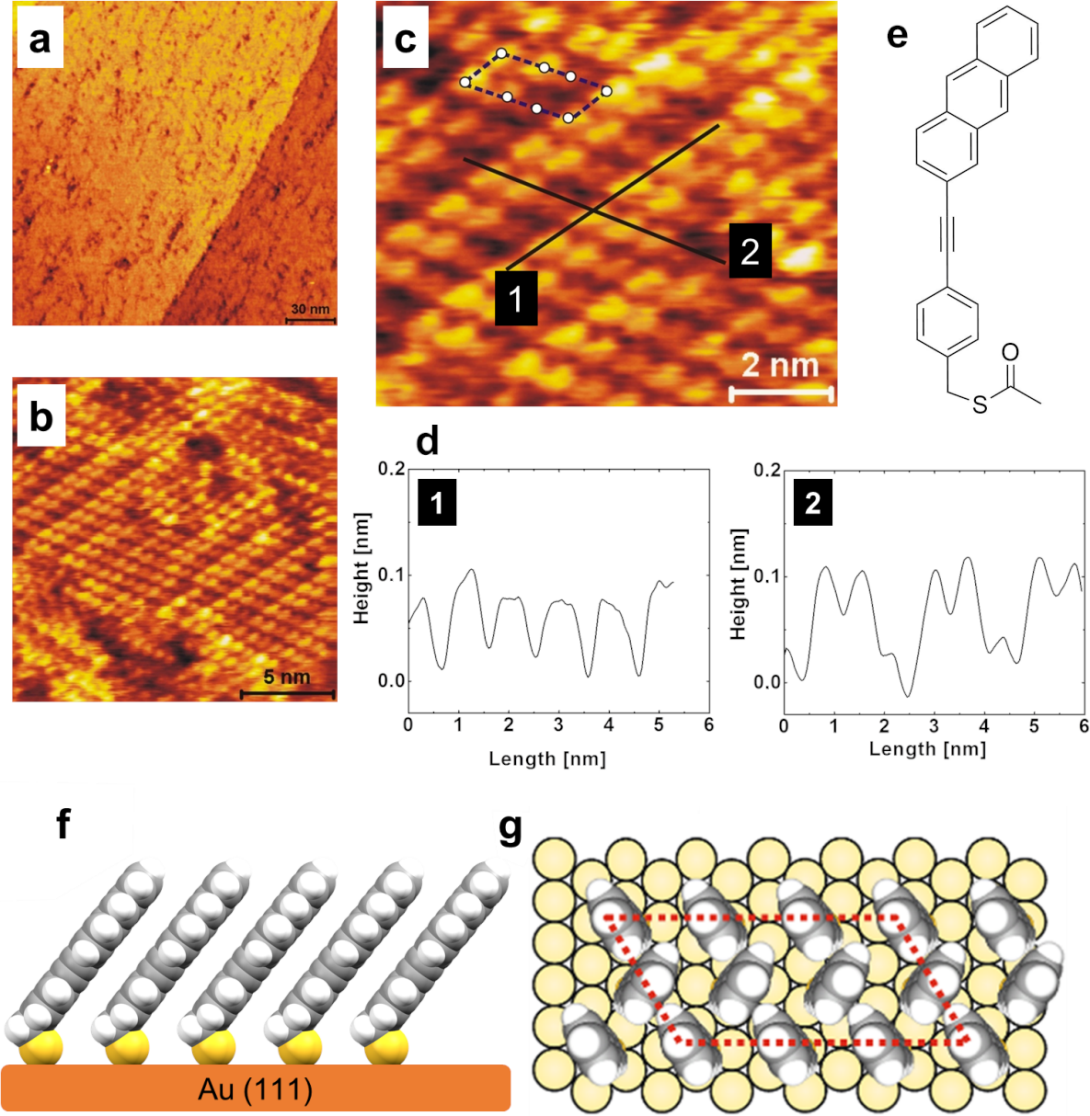
(a), (b) and (c) show STM height images of a PAT SAM on gold at different magnifications describing the molecular structure of the SAM. The unit cell is marked as a parallelogram in (c) with a single domain with the structure (2√3 × 4√3)*R*30°. (d) The height profiles along the two lines in the topography image of (c) labeled with 1 and 2. (e) Structural formula of the compound used to prepare the PAT SAMs, and (f) the schematic side-view illustration of the aligned adjacent molecules in the PAT SAM. (g) A top-view hard sphere structure model of the PAT-SAM.

The main focus of the present paper is on charge transport between adjacent anthracene units for which other reliable data on bulk crystals are available. In the present study, we have used an anthracene unit to which a sulfur anchor atom is connected via a triple bond and another phenyl unit (see Figure 1e). The latter two constituents have shown to have very positive influence on the structural quality of the SAMs [25]. In the present study, two types of organothiols were used. The second organothiol, hexadecanethiol (HDT) is based on alkyl chains and serves as an insulating unit.

## Experimental

### Synthesis of *S*-(4-(anthracene-2-ylethynyl)benzyl) ethanethioate

#### 2-(Trimethylsilylethynyl)anthracene

To a solution of 12 mL of trimethylsilylethyne (85 mmol) in 200 mL of dry THF, 30 mL of a solution of isopropylmagnesium chloride (2.0 mol/L in Et_2_O, 61 mmol) were added under exclusion of oxygen. After stirring the mixture for a couple of minutes to complete the deprotonation, 10 g of 2-bromoanthracene [26] (39 mmol) and 1.36 g of Pd(dppf)Cl_2_ (1.9 mmol) were added and the mixture heated for 5 min. The reaction was allowed to proceed at room temperature for an additional hour (complete transformation was detected by GC–MS), before an aqueous NH_4_Cl solution was added and the product extracted with dichloromethane. The extract was filtered through a pad of silica, evaporated to dryness and recrystallized from methylcyclohexane, yielding 9.1 g (85%) of a yellow solid. ^1^H NMR (CDCl_3_, 400 MHz) δ 8.22 (s, 1H, H-10), 8.20 (s, 1H, H-9), 8.02 (s, 1H, H-1), 7.86–7.82 (m, 2H, H-5, H-8), 7.77 (d, 1H, H-4) 7.34–7.27 (m, 3H, H-3, H-6, H-7), 0.15 (s, 9H, (CH_3_)_3_Si) ppm.

#### 2-Ethynylanthracene

Under exclusion of oxygen, 3 g of 2-(trimethylsilylethynyl)anthracene (10 mmol) were dissolved in 50 mL of THF, before 12 mL of tetrabutylammonium fluoride solution (1.0 mol/L in THF, 12 mmol) were added dropwise. Stirring at room temperature was continued for 1 h and then 50 mL of H_2_O were added. The product was extracted with dichloromethane and filtered through a plug of silica. Evaporation of the volatiles resulted in 2 g of a brownish solid (90%). ^1^H NMR (CDCl_3_, 400 MHz) δ 8.23 (s, 2H, H-10, H-9), 8.05 (s, 1H, H-1), 7.87–7.81 (m, 2H, H-5, H-8), 7.79 (d, 1H, H-4), 7.35–7.28 (m, 3H, H-3, H-6, H-7), 3.04 (s, 1H, CH) ppm.

#### 4-(Anthracen-2-ylethynyl)benzyl alcohol

A mixture of 1.7 g of 4-iodobenzyl alcohol (7.3 mmol), 1.5 g 2-ethynylanthracene (7.4 mmol), 0.282 g copper(I) iodide (20 mol %), and 0.262 g Pd(dppf)Cl_2_ (5 mol %) in 30 mL of triethylamine were stirred under strict exclusion of air at room temperature for 16 h. To improve the solubility of the reactants, 40 mL of dry THF were added and the mixture heated for a short period of time. After another 24 h stirring at room temperature, the mixture was adsorbed onto silica and dried by means of a rotary evaporator. The loaded silica was placed on top of a silica plug and then eluted first with hexanes/dichloromethane 2:1 to remove nonpolar impurities. Then the product was eluted first by dichloromethane followed by warm ethyl acetate. The polar eluates were combined and the volatiles removed in vacuo. The remaining material was recrystallized from chloroform to yield 1 g of a brown solid (44%). ^1^H NMR (DMSO-*d*_6_, 250 MHz) δ 8.63 (s, 2H, H-10, H-9), 8.37 (s, 1H, H-1), 8.17–8.08 (m, 3H, H-5, H-8, H-4), 7.63–7.53 (m, 3H_anthracene_, H-3, H-6, H-7, 2H_phenyl_, H-3, H-5), 7.42 (d, 2H_phenyl_, H-2, H-6), 4.57 (d, 2H, C*H*_2_OH) ppm.

#### *S*-(4-(Anthracene-2-ylethynyl)benzyl) ethanethioate

To a solution of 0.15 g triphenylphosphane (0.57 mmol) in 10 mL dry THF, 0.14 g of diisopropyl azodicarboxylate (0.69 mmol) were added maintaining a temperature of 0 °C. This mixture was added dropwise to a solution of 0.15 g of 4-(anthracen-2-ylethynyl)benzyl alcohol (0.49 mmol) and 0.3 mL of thioacetic acid (4.2 mmol) in 10 mL of THF. The reaction was allowed to proceed for 3 h, before the volatiles were removed in vacuo. The remainder was taken up in a mixture of hexanes and dichloromethane (4:1), and separated by chromatography on silica gel using a solvent gradient starting with the same composition. The product was recrystallized from isopropanol (ca. 100 mL). Yield: 0.10 g of a yellowish solid (61%). ^1^H NMR (CDCl_3_, 250 MHz) δ 8.32 (s, 2H, H-10, H-9), 8.14 (s, 1H, H-1), 7.97–7.87 (m, 3H, H-5, H-8, H-4), 7.48–7.37 (m, 3H_anthracen_, H-3, H-6, H-7, 2H_phenyl_, H-3, H-5), 7.23 (d, 2H_phenyl_, H-2, H-6), 4.03 (s, 2H, CH_2_S), 2.37 (s, 3H, COCH_3_) ppm.

### Preparation of PAT SAMs for STM, conductive AFM and NEXAFS experiments

STM measurements and conductive AFM experiments were carried out on substrates that were prepared by evaporating 180 nm (optionally 300 nm) of Au (99.995%, Chempur) onto freshly cleaved mica (1 × 3 inch, grade V1, TED PELLA, INC.), which was preliminary stored at ≈600 K (optionally 453 K) for 2 days in the evaporation chamber. The thickness and deposition rate (10 Å s^−1^) were monitored using a quartz crystal microbalance. After evaporation of Au, the substrates were cooled and the chamber was backfilled with nitrogen. The substrates were stored under argon and flame-annealed in a butane/oxygen flame immediately before the SAM preparation. The formation of the PAT monolayers was carried out by immersing the substrates into 0.1 mM absolute ethanolic solution (≥99.8%, AnalaR NORMAPUR^®^ ACS, Reag. Ph. Eur. zur Analyse) of PAT for 24 h at RT and, optionally, at an elevated temperature (70 °C). The samples were characterized immediately after the SAM preparation.

For the Near-edge X-ray absorption fine structure (NEXAFS) measurements, gold-coated silicon wafer substrates were used. Gold films of 100 nm thickness were evaporated thermally at 453 K under high-vacuum conditions (≈10^−7^ mbar) with 5 nm titanium as the adhesion layer. The thickness and deposition rate (10 Å s^−1^) were monitored using a quartz crystal microbalance. Between substrate preparation and SAM formation, the substrates were stored in an argon atmosphere. The formation of the PAT monolayers on the gold silicon wafer was described above.

### Grafting experiments

The grafting process was performed with the Bruker Dimension^®^ Icon™ SPM system in a liquid cell filled with a 1 mM ethanolic solution of HDT. The PAT SAM-Au/mica substrate was placed into the solution and allowed to stand for 30 min in order to avoid thermal drift of the sample during grafting. Then, the process of nanografting was performed in the contact mode (cantilever type: NSC-18/Cr-Au, spring const. 2.8 N/m, µmasch, NanoAndMore GmbH) with an increased force (setpoint: −0.66 V) of the tip on the surface. During the scanning process (scan rate: 5 Hz, number of lines: 512), the targeted PAT molecules were removed from the gold surface and instantly replaced by HDT molecules coming from the solution. By this method well defined areas of the existing conductive PAT-based SAM matrix were removed, so that the small conductive PAT islands surrounded by HDT were obtained.

### Characterization methods and instrumentation

All STM measurements were carried out under ambient conditions, using either a Joel JSPM 4210 microscope or an Agilent STM setup, which had been cross-calibrated by imaging HOPG with atomic resolution. The tips were prepared mechanically by cutting a 0.25 mm Pt_0.8_Ir_0.2_ wire (Goodfellow). All data were collected in a constant-current mode with typical tunneling currents of 0.1–0.15 nA and a sample bias of 0.5–0.7 V.

NEXAFS spectroscopy measurements were performed at the HE-SGM dipole beamline at synchrotron facility BESSY II, which is a part of the Helmholtz-Zentrum, Berlin. A detailed description of the experimental setup was given in ref. [27]. The degree of polarization of the recovered radiation was 91% with an energy resolution of about 100 meV at the C K edge. Spectra were recorded at five different angles of incidence of the synchrotron light (θ = 20°, 40°, 55°, 75°, 90°) to determine the molecular orientation.

Infrared reflection-absorption spectroscopy (IRRAS) was performed on a Bruker VERTEX80 spectrometer (Bruker Optics GmbH) by investigating the PAT-SAM on Au/silicon wafers. As a background sample we used a SAM of deuterated octadecanethiol on an Au/silicon wafer.

Time-of-flight secondary ion mass spectrometry (TOF-SIMS) measurements were carried out in a TOF-SIMS 5 device (ION-TOF GmbH, Münster, Germany). The spectrometry was performed in static SIMS mode (primary ion beam dose < 2 × 10^11^ ions/cm^2^) with Bi^3+^ primary ions at 25 keV. Spectra were calibrated on the C^−^, CH_2_^−^, S^−^, and Au^−^ peaks.

The conductivity measurements (PFTUNA probes, spring const. 0.4 N/m) were performed by the PeakForce TUNA^TM^ method of a Bruker’s Dimension^®^ Icon^TM^ SPM system. The scan rate was set to 0.5 Hz, the DC bias to 11.1 mV. Current sensitivity range was set to highest value available (0–100 pA). The current was read out at forces of 90 and 140 nN.

The conductivity data of the grafted islands were evaluated in the following way: (i) in a first step the island diameters were calculated. For that, the AFM current images were used to determine the real surface area A of each island with the particle analysis of the SPIP software (version 6.7.8) by setting the threshold current to 1 pA. (ii) Then – assuming a circular island shape – the island diameter *d* was computed from the area *A* using *d* = 2∙(*A*/π)^1/2^. (iii) Finally the center current of each island was plotted as a function of the calculated island diameter.

## Results and Discussion

First evidence for the contribution of lateral currents to the conductivity in SAMs of conjugated molecules has been found using a STM-based method [18]. Ishida et al. prepared and characterized irregular shaped islands of [1,1′,4′,1′′-terphenyl]-4-ylmethanethiol embedded in an intrinsically insulating 2D matrix of alkanethiolates. When studying islands of different sizes, they found that the apparent island height in STM images increases as the lateral size of domains increases. This effect was explained by contributions from the lateral conductivity in the OSC SAMs, brought about by transfer of charge from the molecule contacted by the STM tip to neighboring molecules. The presence of islands with different apparent height in OSC-based SAMs could be confirmed later for the case of hexabenzocoronene (HBC)-thiolates by Käfer et al. [28]. They confirmed that the π–π coupling of adjacent molecules allows for a lateral intermolecular transport of the electrical charge within the HBC islands, resulting in a higher conductivity and a different apparent height in the STM depending on the island size. However, in the work of Käfer et al. the apparent height (i.e., the conductivity) flattened out for increasing island sizes, apparently approaching an asymptotic value. This is unexpected, increasing the island sizes should lead to an increase of conductivity, and thus of the apparent height.

The presence of substantial contributions from lateral conductivity within self-assembled monolayers was further corroborated by Bashir et al. through the application of molecular dynamics simulation where the charge transport was taken explicitly into account [29]. The theoretical study on the optimized model of HBC by using the Ehrenfest (mean field) approach demonstrated that the molecular packing of the monomers within the SAM is beneficial to the intermolecular electronic coupling and further promote charge carrier mobility. In accordance with the simulation, the experimental analysis of the apparent height of the islands as a function of island diameter in the SAMs yielded a rather high charge carrier mobility of 6.7 cm^2^·V^−1^·s^−1^.

Although these studies represent a major step forward with regard to determining intrinsic charge carrier mobilities in organic semiconductors, it has to be noted that in this previous approach the conductive islands were formed in a random process, which made a control of their size and shape virtually impossible. Because of the irregular shapes of the conductive islands, a thorough analysis of the experimental data is very difficult. In order to avoid these shortcomings, and to further understand the mechanism of the lateral current transport, we have developed a new strategy using an AFM-based lithography [30]. In the context of this new approach, the so-called “nanografting” method is used to “write” differently sized regions of OSC-containing thiolates embedded into an insulating SAM matrix. Furthermore, since the determination of apparent heights of islands from STM data is somewhat indirect, the conductivities of the patterned areas were determined directly by means of highly sensitive current measurements, where the conductive tip of the AFM is used as a top electrode. This setup allows determining the topography and the conductivity of the SAM simultaneously. In course of this project, the relationship between island size and conductivity could be fully confirmed, and the lateral charge transport and carrier mobility of the semiconductor molecules were determined from the dependence of conductivity on the island size.

In the present study, we used PAT protected with a thioester group to fabricate OSC-based SAMs. Anthracene is one of the best-studied organic semiconductor molecules, and numerous studies on charge-transport within single-crystals made from this compound have been reported [31]. A high mobility of charge carriers has been observed in field-effect transistors (FET) made from this compound [32]. Anthracene-2-thiol, obtained by functionalization of anthracene with a thiol group, was used to fabricate SAMs on the top of Au bottom electrodes, which resulted in a beneficial effect on the performance of field effect transistor devices using pentacene as organic semiconductor [33]. In that work, the increase in performance was attributed to the reduced sheet resistance for charge transport in the anthracenethiol monolayer supporting the pentacene multilayer.

In order to study the *I*–*V* characteristics of differently shaped islands within a PAT SAM, first PAT-layers were deposited on the Au(111) surface by immersing Au substrates into ethanol solution of PAT protected with a thioester group. Deposition was carried out at 70 °C. At this elevated temperature the thioester protective group was removed [34], and after immersion times of about 12 hours a well-defined SAM had formed on the surface. The as-prepared SAMs were analyzed by ToF-SIMS. As expected, the main contribution in these spectra was a molecular ion peak at *m*/*z* 323.1, assigned to a C_23_H_15_S species (Supporting Information File 1, Figure S1). Further characterization methods such as infrared reflection absorption spectroscopy (IRRAS) and attenuated transmission reflection (ATR) spectroscopy were also applied. The assignment of the molecular vibrations was aided by quantum-chemical calculations, from which the transition dipole moments (TDM) of the respective vibrations can be identified and used for an estimation of the orientation due to the surface selection rule in IRRAS. For comparison, the IRRA spectrum of the PAT-SAM, ATR spectrum and Gaussian IR spectrum for PAT were recorded and evaluated (Supporting Information File 1, Figure S2). The absorption signals originating from PAT are clearly observed in the spectral region of 800–1700 cm^−1^. The result corresponds well to the previously reported spectra for anthracenethiolate (Ant-S) SAMs on gold [35] and anthraceneselenolate (Ant-Se) SAMs on gold [36]. The detailed assignments of the bands were done on the basis of previous reports and are summarized in Supporting Information File 1, Table S1. Similar absorption peak positions in bulk and SAM spectra indicate the successful preparation of ordered PAT layers on the Au surface. Further intensity comparisons of the peaks in these two spectra are in accordance with the assumption of monolayer formation [26]. Note that a striking difference between the bulk and the PAT-SAM spectra is that the vibrational peaks at 942 cm^−1^ and 889–893 cm^−1^ for CH/CC vibrations of anthracene present in ATR spectra become a broad peak at 850–1000 cm^−1^ in the IRRA spectra, which means the attenuation of the aromatic out-of-plane bands is apparent for PAT-SAMs. The manifested broad peak can be attributed to the presence of phenyl units with a slight tilt angle and anthracene units with a near vertical orientation. The IRRA spectra show the in-plane bands (most notably 2–3 and 5 in Table S1, Supporting Information File 1) and are still visible and even look enhanced in relative intensity, which means the TDMs oriented parallel or almost parallel to the anthracene framework. Thereof, it can be assumed that the anthracene moieties should be oriented almost perpendicular to the substrate surface and the phenyl groups are tilted to the substrate surface.

The quantitative analysis of near-edge X-ray absorption fine structure (NEXAFS) data (Supporting Information File 1, Figure S4) reveals that the anthracene units within the SAMs exhibit an orientation of the aromatic anthracene cores with an inclination angle almost perpendicular (80° relative to the surface plane) to the surface, whereas the phenyl ring is tilted by 73° relative to the surface. The overall tilt of the molecular axis relative to the surface normal amounts to about 55–60°. It is noteworthy that the differences in the apparent tilt angles of the phenyl and anthracene units may be a result of different intramolecular twist angles. In particular, the nonsymmetrical attachment of the anthracene unit is known to result in a more upright orientation independently of the orientation of the spacer group [26].

In a next step, STM images were recorded that demonstrate SAM formation of PAT on an atomically smooth Au(111) surface. As shown in the control STM data (Supporting Information File 1, Figure S3), the initially distributed smaller nucleation centers with a leaf-like structure grow together gradually and finally produce the homogeneous PAT SAMs. The molecules in the closed homogeneous PAT SAMs line up and are well aligned within a domain (Figure 1). This observation confirms a previous report on anthracene-based SAMs made from a different anthracenethiol, were the –SH group was directly attached to the anthracene, yielding SAMs with the anthracene unit much closer to the surface [35]. The individual domains within the SAMs show three different orientations. From high-resolution data the determination of the unit cell (see drawn parallelogram with *a* = 0.95 ± 0.05 nm and *b* = 2.15 ± 0.1 nm) which goes best in line with a (2√3 × 4√3)*R*30° cell.

This cell has an area of 1.73 nm^2^ within which three elongated protrusions could be found, suggesting a surface area per molecule of 0.577 nm^2^. This would imply a relatively large tilt angle of the molecules and would not go in line with the almost upright molecular orientation determined from NEXAFS. We thus conclude that each of the protrusions is caused by two molecules, reducing the area per molecule to 0.287 nm^2^, which is the same value as found for anthracene-2-thiolate monolayers (0.287 nm^2^) [35]. The strong variation of apparent molecular heights within each unit cell has to be explained by a lifting of the potential inversion symmetry of this cell due to the tilt of the molecules.

**Figure 2:**
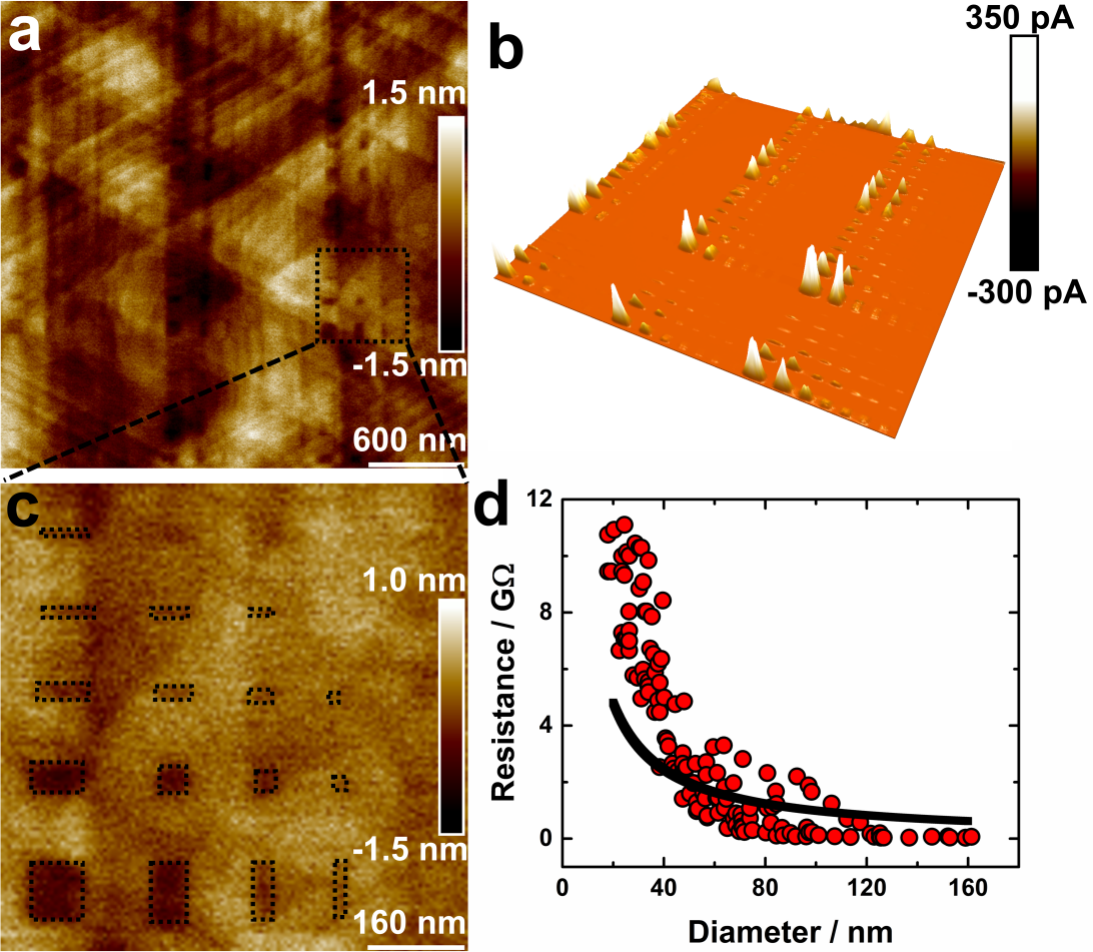
(a) Topography and (b) the corresponding 3D current image (DC bias: 11.1 mV, current sensitivity range: 0–100 pA) of islands obtained from HDT matrix inserted into the PAT SAM. (c) Enlarged topography view of different islands which are marked with a dotted frame shown. (d) Resistances measured for different PAT islands as a function of island diameter. The black line shows the result of a fit in the diameter interval 20–160 nm by using Equation 1.

In order to yield PAT SAM islands of well-defined size and shape embedded in an insulating 2D matrix made from another thiol, we used the nanografting technique introduced by G.-y. Liu et al. [37]. HDT was used as the insulating thiol to decouple the PAT islands. Starting from a high quality PAT SAM, in well-defined PAT areas the OSC-based thiol was replaced by HDT using nanografting carried out in a liquid cell of an AFM. Using this procedure, we were able to prepare precisely defined islands of conductive PAT SAMs embedded into an insulating HDT matrix, see Figure 2a. Obviously, the patterns created by nanografting provide islands which are much better defined than the irregularly shaped, randomly generated conductive islands used in previous works [18,28]. Note also that the nanografting of the insulating HDT into the previous fabricated PAT SAMs with high structural quality ensures that the density of defects within the OSC islands is very low.

Previous STM investigations of HBC-thiolate islands embedded in a surrounding decanethiolate matrix [28–29] showed different apparent heights, which depended on the diameter of the islands. The apparent height depends on both, the geometric height and the current density [38]. The extraction of conductivities from these data on apparent height, however, is rather indirect. In contrast, with the conductive AFM the conductivity can be determined for a fixed height of the AFM tip above the SAM surface directly from simultaneous measurements of the topography and conductive response of the SAMs.

The topography and the associated conductivity measurements of the sample surface with PAT islands prepared by grafting insulating HDT in the PAT SAM are shown in Figure 2. Figure 2a and Figure 2c show the topography image of a representative surface area with grafted PAT islands with different magnifications. Figure 2b represents the 3D image of the current corresponding to Figure 2a. As shown in Figure 2c, PAT islands surrounded with HDT can be recognized as dark depressions. The height difference of the two different SAM areas measured by AFM amounts to 0.50 ± 0.05 nm, which compares well with the theoretical height difference expected from the difference in length of the two SAM-forming molecules (1.68 nm for PAT, according to van der Waals structures [39] and 2.3 nm for the HDT [40]). The electrical conductivity as determined from the *I*–*V*-curves measured with the conductive probe AFM increases with the calculated diameter of the PAT islands (Figure 2d). The grafted patterns are clearly identified in the conductivity maps and are shown as areas marked with dotted lines in Figure 2c. As expected, the conductivity of the islands is much higher than that of the HDT region. While for PAT islands – depending on their size – currents of up to 509 pA at a voltage of 11.1 mV were observed, the maximum current for the HDT areas amounted at this voltage was so small that it could not be measured (i.e., *I* < 0.1 pA). On pristine PAT areas (prior to any AFM-based lithography), very large currents in excess of 5 nA were measured at the same voltage (11.1 mV). This observation reveals that the pristine PAT SAM exhibit ordered regimes in which the lateral conductivity is not affected by defects etc., and thus is substantially larger than the rectangular patterns created by AFM lithography. The presented preparation of PAT islands with arbitrary sizes and shape structures can be used to more thoroughly investigate the dependence of conductivity on the size of the PAT islands. In order to carry out a thorough evaluation, a fairly large number, 134, of different PAT islands surrounded by insulating HDT areas were fabricated and subsequently characterized. The currents measured for each island are plotted as a function of the calculated island diameter of the corresponding PAT islands, as shown in Figure 2d. These data reveal a substantial and continuous increase in current with increasing surface area. Interestingly, no asymptotic value is reached for the sizes of patterned areas studied here. This is in contrast to the observations reported in previous work for spontaneously formed islands [28] for which the conductivity showed an asymptotic behavior with increasing island size. We explain this behavior by the higher structural quality of the islands used in the present work. In the work of Käfer et al. [28] the formation of the conductive islands was a spontaneous process. As soon as the insulating decanethiolate SAM was exposed to the solution containing the conductive HBC molecules, circular HBC islands with sizes limited to a few nanometers were formed. This process is expected to lead to rather small 2D domains. Also the exchange of the decanethiolate moieties by HBC may be incomplete, which would also lead to a larger defect density. We speculate that the asymptotic behavior of conductivity increase with island size results from a fairly large amount of defects in the islands formed by the exchange process. For larger island sizes such effects could limit the further increase of lateral conductivity.

In contrast, the PAT islands used in this work essentially have the high structural quality of the original SAM, since we used AFM lithography to “insulate” them from the surroundings by grafting HDT onto the surface. As a result, conductive PAT islands with different sizes remain on the Au surface, separated by the insulating barriers consisting of HDT. It is an important benefit of this approach that the structure of the PAT molecules within the islands should be identical to that of the continuous SAM formed in the previous step. We expect this fact to lead to increased domain sizes and thus to a smaller density of defects within the islands.

Note that all these measurements were carried out with constant force applied to the AFM tip. Accordingly, we assume that the contact area is the same for all measurements carried out on PAT islands larger than a critical size when the tip was positioned in the middle of the island. In order to obtain an upper limit for this critical size, we have analyzed the apparent size of the main boundaries and other defects in the AFM micrographs. On the basis of this analysis, we estimate that the diameter of the tips must be smaller than 20 nm. We thus assume that all measurements carried out with the tip positioned in the center of an island with an island diameter of larger than 20 nm are not influenced by tip size effects.

**Figure 3:**
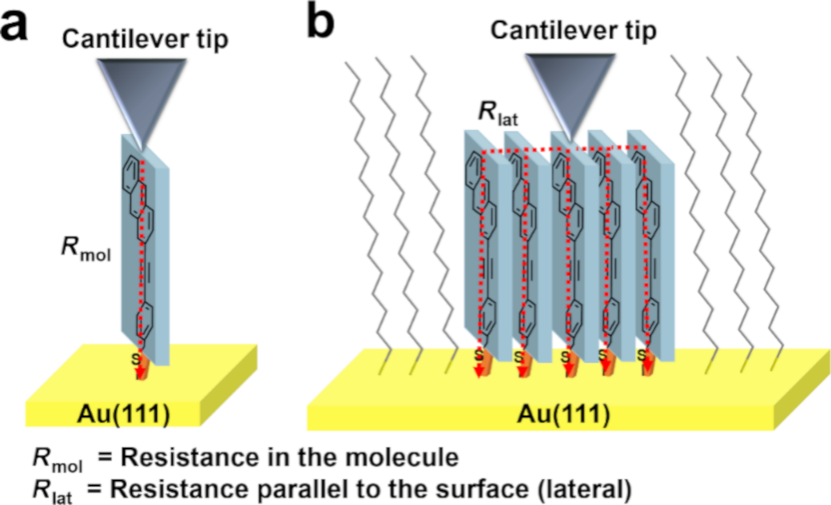
a) Simple model of resistance within a single thiol molecule on the gold(111) surface. b) Model of charge transfer within a continuous SAM. The number of parallel resistors depends on the island size. The PAT is shown schematically in blue with the S anchor group on a gold surface, while the respective resistances are shown laterally and within the thiol.

The schematics presented in Figure 3 rationalize the observation that the conductivity increases with island size. In the hypothetical case that the AFM tip contacts only a single PAT molecule, i.e., an island consisting of a single molecule only, we would obtain the resistance of a single molecule. Such values have been reported in the literature, e.g., for TP1 this value amounts to 55.5 MΩ [41]. For larger islands, in addition to the vertical current through one single molecule, additional current (lateral) pathways through adjacent molecules are available. While for HDT the conductivity between adjacent monomers is very low, Figure 2 clearly reveals that this effect is present in PAT islands. For the same voltage, the current rises by two orders of magnitude when the size of the island is increased from 20 nm (smallest island) to 160 nm (biggest island). This strong current increase clearly confirms the dependence of PAT island conductivity on their lateral size and thus unequivocally cooperates with the presence of pronounced lateral conductivity in PAT islands.

This observation can be explained by the model depicted in Figure 3, which has been described in previous publications [28]. Under the assumption that the contact resistance between the AFM tip and the PAT molecule or the PAT island is so small that it can be neglected, the resistance is only due to the molecular resistance *R*_mol_ of the thiolate itself. While adjacent HDT molecules do not contribute to the overall current, PAT islands will increase the total current due to the intermolecular charge transfer between neighboring molecules. For the hypothetical case of an island consisting of three molecules, with the AFM forming a direct contact to the center molecule only, we would yield a resistance of


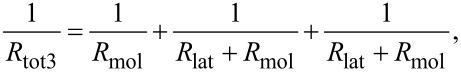


where *R*_lat_ is the resistance between two adjacent molecules.

For larger islands, we use an expression established previously [29], where the total resistance *R*_netw_ amounts to:

[1] $$R_{\text{netw}}=\frac{R_{\text{mol}}\left(R_{\text{mol}}+\frac{n}{3}R_{\text{lat}}\right)}{\frac{n}{3}R_{\text{lat}}+R_{\text{mol}}\left(n+1\right)}$$

with *n* being correlated to an island width via *n* = width/0.8 nm as obtained from unit cell dimensions in high-resolution STM images (see Figure 1). This expression is not strictly correct, but numerical simulation reveals a deviation of less than 1% [29]. In the following we assume a 1D transport of charges along the (112) direction, because from the packing of the PAT-molecules (see Figure 3 and Figure 1g), we expect good π–π-overlap only along the (112) direction.

The values *R*_mol_ and *R*_lat_ were determined by fitting the experimental resistance vs island diameter curve with the formula provided in Equation 1 (see Figure 2d). Note that the fit is not very good, indicating that the 1D model used for the derivation of Equation 1 (conductivity only in one direction) is too simple for the present PAT SAM. In any case, the value obtained from the fit is consistent with previous studies. For the resistance *R*_mol_ of a PAT molecule (governing the vertical current through a single molecule) we yield a value of 142.1 MΩ. This value is somewhat larger than those reported in previous literature [36], e.g., for TP1, a value of 55.5 MΩ has been reported [41]. Since PAT is longer than TP1, the larger value for PAT appears reasonable.

From the fit of the data shown in Figure 2 we yield a fit value of *R*_lat_ = 4.28 MΩ for the lateral resistance between adjacent PAT-thiolates. This value is in very good accord with previous work, where values of 2 MΩ and 1.5 MΩ have been reported for HBC_C3 and HBC_Ph, respectively [28]. We explain the larger resistance for PAT by the smaller size of the aromatic core (anthracene vs HBC).

Finally, we relate the lateral resistance, *R*_lat_ to the charge carrier mobility of anthracene and the charge carrier density *N* using the relation


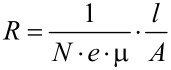


Here, *l* denotes the length of the current path, and *A* the cross-section. Using an average e-mobility in anthracene single crystals of 1 cm^2^/V s at 300 K [42], and a value of l/A of 1 nm^−1^, we yield a charge carrier concentration of ≈1 × 10^19^ cm^−3^. Using a molecular volume of about 1 nm^3^, this value corresponds to about 0.01 electrons per molecule. This rather low value is fully consistent with the fact that the *I*–*V* curves (see Supporting Information File 1, Figure S5) reveal that we are clearly in the linear regime, i.e., currents are clearly below the regime affected by space-charge effects. Such effects come into play at values of 0.1 e/molecule [28]. We thus conclude that the lateral resistance determined from our analysis is fully consistent with the reported electron charge carrier mobility of anthracene and a charge carrier concentration of 1 × 10^19^ cm^−3^.

## Conclusion

In conclusion, we demonstrate a novel approach for measuring charge transport in well-defined self-assembled monolayers, SAMs, containing aromatic cores. Using AFM-based lithography, islands of regular shape are carved out of regular SAMs. These patches are then isolated from the surrounding monolayer by insulating stripes made of a large band-gap alkanethiolate SAM. Measurements using a conductive probe AFM yield a pronounced dependence of current on island size, from which a lateral resistance coupling of adjacent anthracene cores of 4.28 MΩ can be determined. Assuming a bulk charge carrier mobility of 1 cm^2^/V·s, this value corresponds to a low charge carrier density of 0.9 × 10^19^ cm^−3^. Such a low value is fully consistent with the fact the *I*–*V* curves indicate that the currents used here are well below the space-charge limited regime.

## Supporting Information

The supporting information contains (1) Figure S1 with the ToF-SIMS spectra of PAT and HDT SAM on gold/silicon wafer substrates, (2) Figure S2 with the IRRA spectrum of the PAT-SAM, the ATR spectrum and Gaussian IR spectrum of PAT, (3) Table S1 summarizing the assignment of the most intense bands in the calculated, bulk, and monolayer IR spectra of the PAT, (4) Figure S3 containing the topography STM images of PAT SAMs on a pure gold surface, (5) Figure S4 with the results of NEXAFS measurements of the PAT SAMs, (6) Figure S5 with the *I*–*V* curves of the pristine PAT SAM and rectangular SAM patterns of different size, and (7) Figure S6 showing the currents measured for different PAT islands.

File 1 Additional figures and tables.
