# Supplementary material for: Mobility of charge carriers in self-assembled monolayers
Source: Beilstein J Nanotechnol. 2019 Dec 11;10:2449–58. doi: 10.3762/bjnano.10.235 (PMC6941449; doi:10.3762/bjnano.10.235)
Supplement: File 1 — Additional figures and tables. [file Beilstein_J_Nanotechnol-10-2449-s001.pdf]

BEILSTEIN JOURNAL OF NANOTECHNOLOGY

Supporting Information

for

Mobility of charge carriers in self-assembled monolayers

Zhihua Fu, Tatjana Ladnorg, Hartmut Gliemann, Alexander Welle, Asif Bashir,
Michael Rohwerder, Qiang Zhang, Björn Schüpbach, Andreas Terfort and Christof Wöll

Beilstein J. Nanotechnol. **2019,** *10,* 2449–2458. doi:10.3762/bjnano.10.235

Additional figures and tables

License and Terms: This is a supporting information file under the terms of the Creative Commons Attribution License (https://creativecommons.org/licenses/by/4.0). Please note that the reuse, redistribution and reproduction in particular requires that the authors and source are credited.
The license is subject to the *Beilstein Journal of Nanotechnology* terms and conditions: (https://www.beilstein-journals.org/bjnano)

Contents

Figure S1. ToF-SIMS spectra of PAT and HDT SAM on gold/silicon wafer substrate. S2

Figure S2. a) The IRRA spectrum of the PAT-SAM, ATR spectrum and Gaussian IR spectrum of PAT.3S

Table S1. Assignment of the most intense bands in the calculated, bulk, and monolayer IR spectra of the PAT S4

Figure S3. Topography of STM measurements on SAM formation of PAT on the pure gold surface S5

Figure S4. NEXAFS measurements of the PAT SAMs S6

Figure S5. I/V-curves of the pristine PAT SAM and rectangular patterns of different size S7

Figure S6. Currents measured for different PAT islands. S8

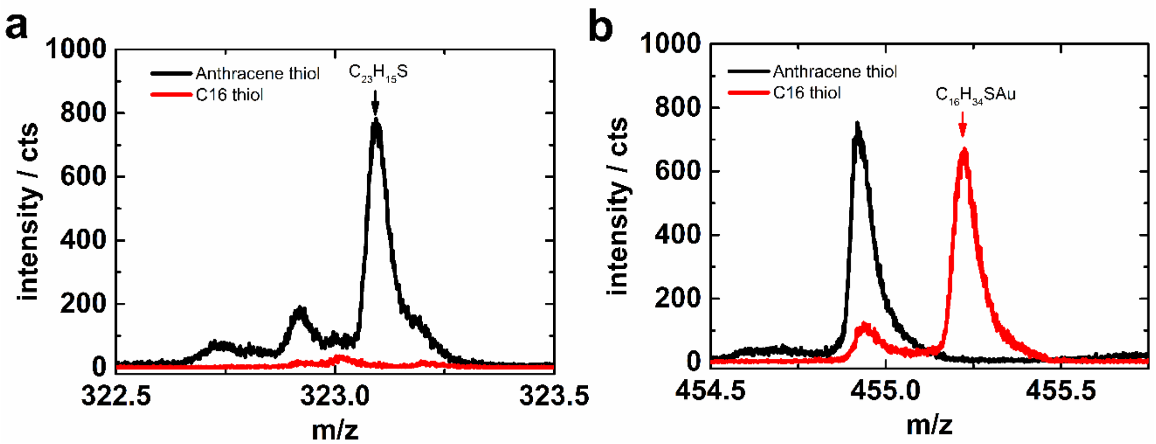

Figure S1. ToF-SIMS spectra obtained from the PAT (black) and HDT (red) SAM on gold/silicon wafer substrate. Both SAMs used in this study exhibit characteristic quasi-molecular signals in SIMS. In case of PAT a signal at 323.102 m/z (labeled with black arrow in (a)) can be assigned to $C_{23}H_{15}S^-$, (323.089 m/z theo.). In case of the HDT besides the $[M - H]^-$ signal, $C_{16}H_{33}S^-$, at 257.245 m/z (257.230 m/z theo.), a more prominent peak is recorded at 455.237 m/z (labeled with red arrow in (b)) assigned to $C_{16}H_{34}SAu^-$, (455.205 m/z theo.).

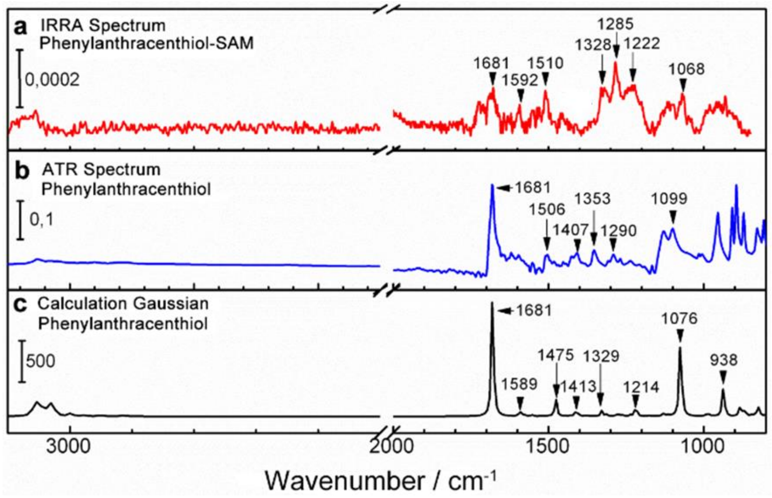

Figure S2. a) The IRRA spectrum of the PAT-SAM, b) compared to the measured ATR spectrum and c) Gaussian IR spectrum for PAT.

Table S1. Assignment of the most intense bands in the calculated, bulk, and monolayer IR spectra of the PAT[a]

Band	assignment	TDM[a]	Calculation/ cm^{-1}	ATR / cm^{-1}	IRRAS/ cm^{-1}
**1**	ν C=O (protecting group), or. ν C=C-(stretching)		1681	1681	1681
**2**	ν C=C stretching , CH bend	ip	1589		1592
**3**	ν C=C stretching, δ CH bend, C-C-C bending (aromatic skeleton vibration) (m)	ip	1475	1506	1510
**4**	δ CH_3		1413	1407	-
**5**	ν C=C stretching, δ CH bend, δ Ring vibrations of anthracene	ip	1329	1352	1285,1328
**6**	CH_2 vibrations (wagging)		1214	1290	1222
**7**	CH_3 vibrations (wagging)		1076	1099	-
**8**	CH, scissor (out of plane)	op	938	942	850-1000 broad peak
**9**	CH, wagg. (out of plane)	op	889-893	889-893	-

[a] Orientations of the transition dipole moment (TDM) of sample modes are listed in the third column. ip, TDM is fully or almost in plane of the aromatic rings; op, TDM is mainly or fully perpendicular to the plane of the aromatic rings.

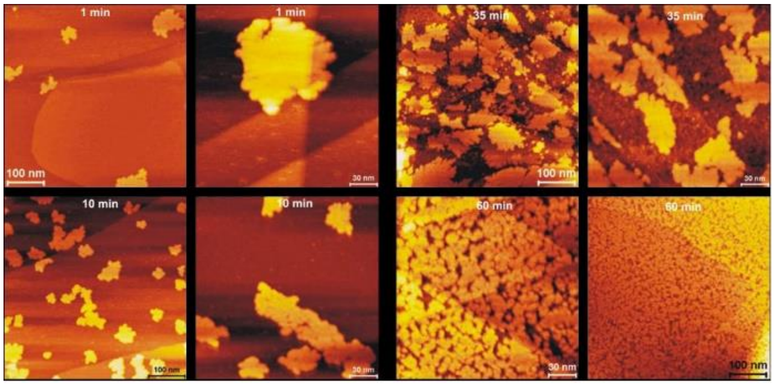

Figure S3. Results of topography of STM measurements on SAM formation of PAT on the pure gold surface, at different magnifications and different incubation times. The gold surface was examined after incubation times of 1 minute, 10, 35 and 60 minutes each with the STM. Topographical images showing an overview of the surface and an enlargement of the imaged PAT islands are shown.

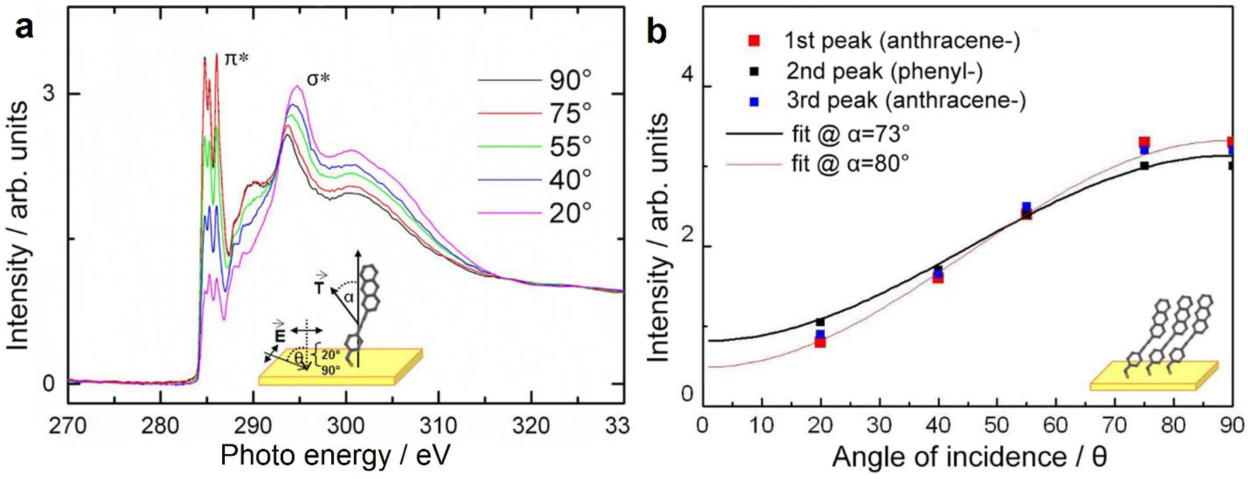

Figure S4. a) Angular-dependent NEXAFS measurements of the PAT SAMs on gold surface to determine the orientation of the thiol on the surface. b) NEXAFS Measurements of the PAT SAMs to determine the orientation of the molecules on the gold surface.

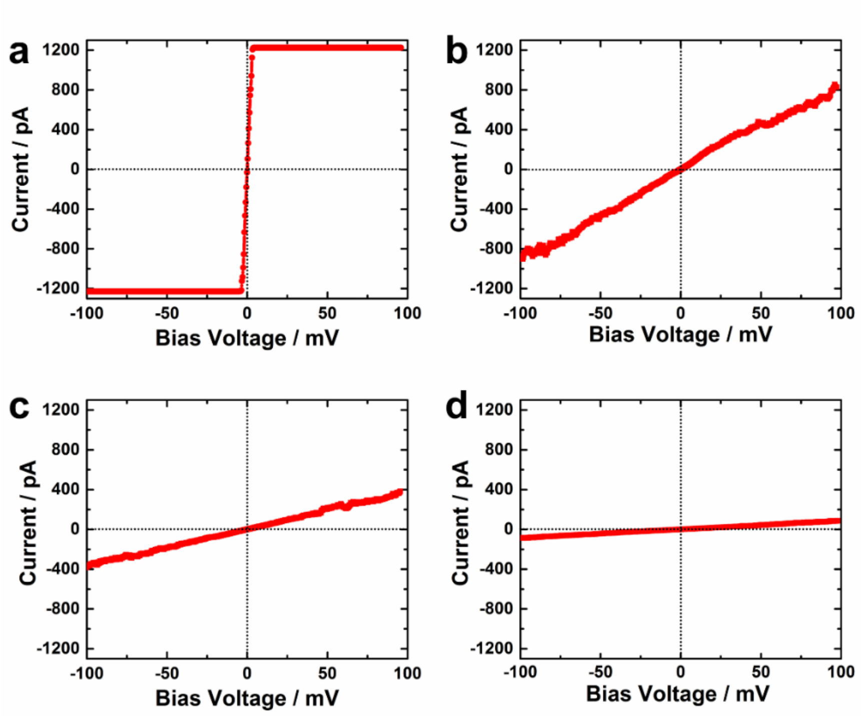

Figure S5. I/V-curves of a) the pristine PAT SAM and rectangular patterns of different size (b: 7989 nm^2, c: 3373 nm^2 and d: 1295nm^2). The current at a bias voltage of 11.1 mV is (b) 92.4 pA, (c) 40.6 pA and (d) 9.2 pA, respectively.

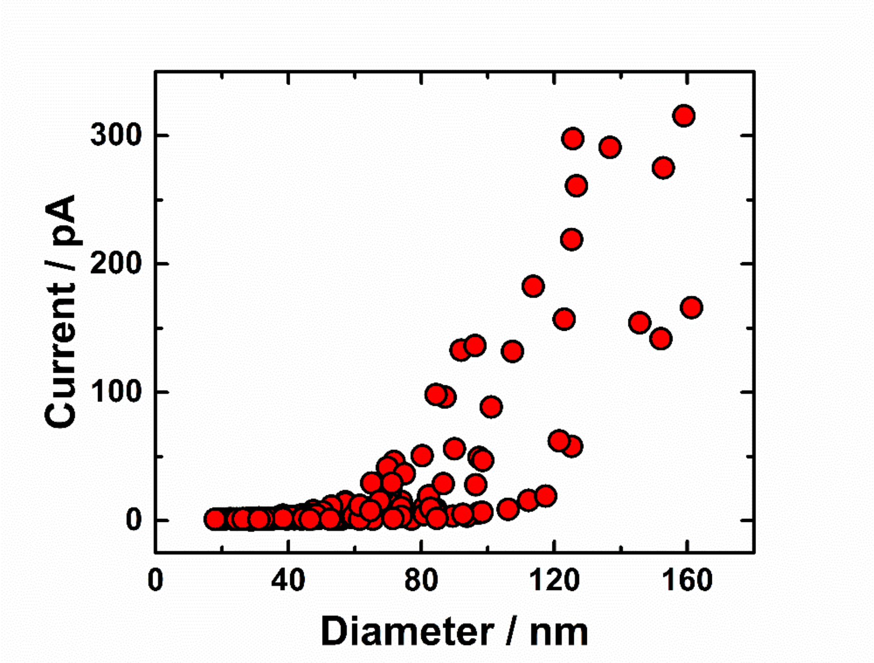

Figure S6. Currents measured for different PAT islands as a function of the square root of the island surface area.
